# Trends of Human Metapneumovirus Outbreaks in Bangladesh: Probable Risks and Preventive Strategies

**DOI:** 10.1002/hsr2.71300

**Published:** 2025-09-22

**Authors:** Nadim Sharif, Shuvra Kanti Dey

**Affiliations:** ^1^ Department of Microbiology Jahangirnagar University Savar Dhaka Bangladesh; ^2^ CMB Program University of Vermont Burlington Vermont USA

**Keywords:** Bangladesh, human metapneumovirus, outbreaks, preventive policies

## Abstract

**Background and Aims:**

Human metapneumovirus (hMPV) is one of the major pathogens associated with respiratory tract infection and is a rising concern for people with compromised immunity worldwide. Amid the global concern about the increased incidence of hMPV, we found a significant lack of studies on the epidemiology of this virus in Bangladesh.

**Methods:**

In this study, we integrated the previous reports of hMPV in Bangladesh and characterized the cases.

**Results:**

We found that hMPV has been continuously circulating since 2000 among the residents of Bangladesh. The prevalence varied from 3.9% to 33% among children aged below 5 years. The cases were reported from Dhaka, Chittagong, Cumilla, Bogura, and Barishal. Among the symptoms, fever (80%–100%), cough (85%–100%), tachypnea (70%–100%), runny nose (80%–97%), and difficulty breathing (70%–85%) were the most common.

**Conclusion:**

This is the first integrated study of hMPV outbreak characterization in Bangladesh.

## 1 Introduction

Increased cases of 2024‐human metapneumovirus (hMPV) infection have been reported from China, Japan, Malaysia, and India. Though first discovered in 2001 in the Netherlands, hMPV was reported worldwide within the next 5 years [1, 2]. It is the second most prevalent virus infecting the respiratory tract after RSV. It is said that everyone gets infected at least once with hMPV in their lifetime [3]. It is a member of the family Pneumoviridae with a single‐stranded negative‐sense RNA genome of 13 Kb. The hMPV has a close evolutionary relation with the avian metapneumovirus [4]. The genome of hMPV encodes for 9 proteins in an orientation like 3′‐N‐P‐M‐F‐M2 (M2–1/M2–2)‐SH‐G‐L‐5′. The G and F proteins are crucial for binding to human epithelial cells and the multiplication of the virus [4].

The hMPV cases have been reported in about 10%–15% of the acute respiratory tract illnesses among children in the USA. About 15% of the cases required hospitalization [5]. However, until the 2024 hMPV outbreak, the scattered reports were not alarming and were regarded as seasonal incidence. A significant proportion of cases (6.2% proportionate incidence) and hospitalizations (5.4% proportionate incidence) of acute respiratory tract illness were linked to hMPV, surpassing COVID‐19, adenovirus, or rhinovirus in China. A higher number of cases with a higher growth rate than previous outbreaks have also been noted in Hong Kong and Malaysia in 2024 [2]. On the contrary, the link between these cases across different countries has not been elucidated with the present knowledge.

Depending on the characteristics of F and G genes, hMPV is classified into four genotypes, namely A1, A2, B1, and B2, and further classified into several lineages (A1, A2.1, A2.2.1, A2.2.2, B1, and B2) [6]. Though cocirculation of different sublineages in the same population is frequently reported, the association of any particular genotypes or sublineage with severe symptoms is poorly characterized. Evolutionary analysis supports the presence of hMPV in humans for the last 50 years [3, 6]. The most probable spread occurs from infected individuals to the susceptible via droplets, close personal contact, and fomites contaminated with virus‐containing droplets produced during coughing, talking, sneezing, or laughing [3, 7]. The recent outbreaks and increased sporadic cases of hMPV in several countries in South‐East Asia and China have become a health concern. New cases have been reported in Bangladesh recently. However, the outbreak surveillance and epidemiologic studies of hMPV are lacking worldwide. On top of that, few studies have been conducted before with poor sociodemographic characterization and case presentation. We conducted this study to determine the recent trends of an integrated overview of previous reports of hMPV outbreaks in Bangladesh.

## 2 Human Metapneumovirus in Bangladesh

**Figure 1:**
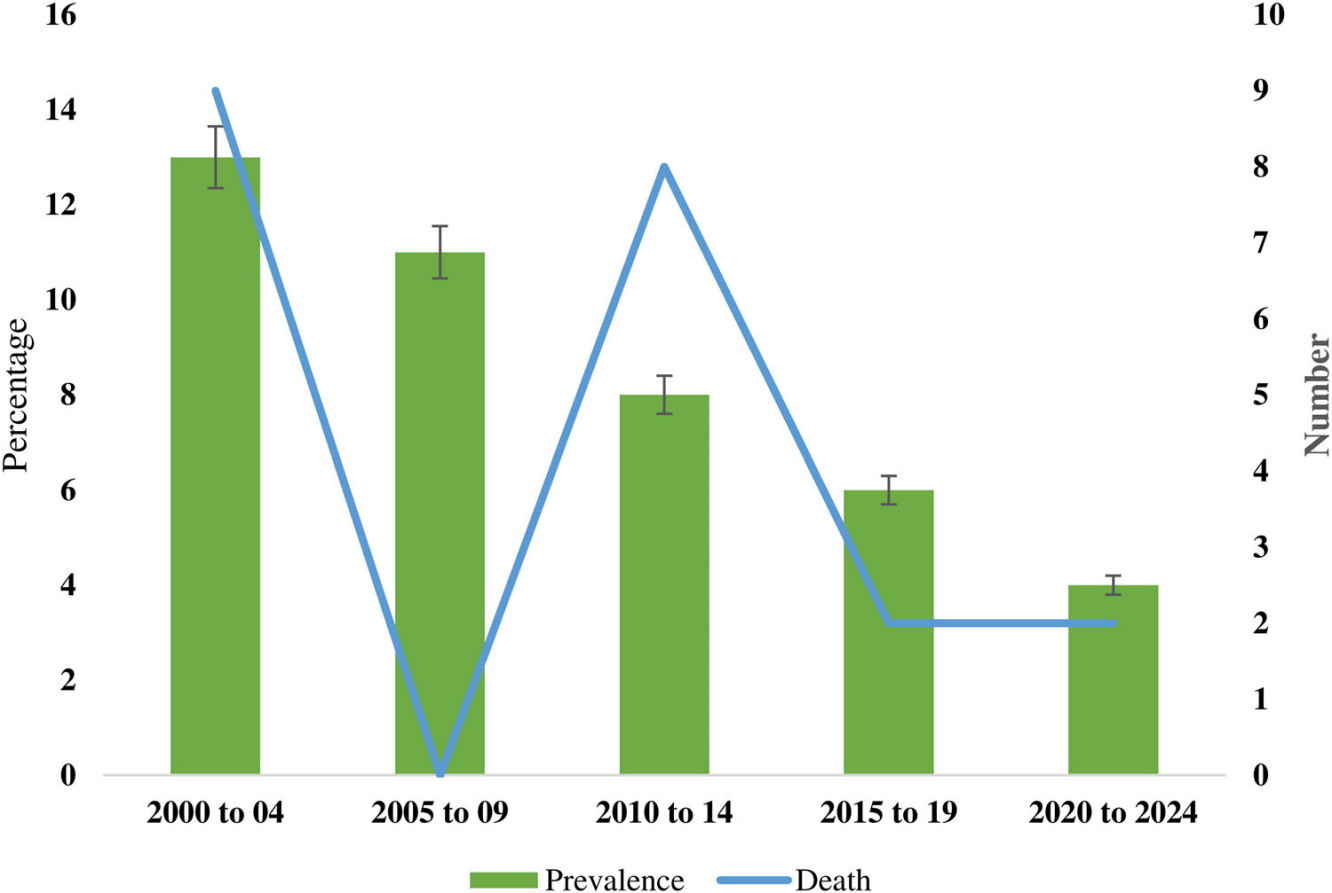
Prevalence and mortality of hMPV in Bangladesh during 2000–2024.

Amid an increased growth of infection rates in China, Japan, and Malaysia, and reports of new cases from India, the first case of 2024‐hMPV was reported from Bangladesh on January 9, 2025 [8]. The infected individual was a 30‐year‐old woman, and RT‐PCR confirmed the case. After receiving treatment for 9 days in the hospital, she died on January 16, 2025. She was further diagnosed with *Klebsiella pneumoniae* (*K. pneumoniae*) infection and developed symptoms including fever, cough, chills, shortness of breath, extreme tiredness, and chest pain. The case had no recent travel history outside the country [8]. However, her contact history before developing the symptoms and after confirmation of the hMPV is still unknown. The first detection of hMPV was done in 2001 in an urban community in Dhaka [9]. Cases of hMPV in children under 5 years have been reported regularly with a prevalence ranging from 4% to 33% since 2001 (Figure 1). Most cases were reported from Dhaka, and a single study included children from Barishal, Bogura, Kishorganj, and Cumilla (Table 1). Another recent study of the migrated Rohingya people in Chattogram also found cases of hMPV in 3.9% of the study children. Most of the study participants were analyzed with symptoms of acute respiratory tract illness or pneumonia and coinfection with other viruses. Both genotypes A and B are circulating with peak incidence in January, May, and September. Two deaths in children aged < 1 year were reported in 2012 due to hMPV infection.

**Table 1:** Trends of human metapneumovirus infection in Bangladesh.

Year	Age of the infected individuals	Prevalence, % (*n*)	Regions	Other notable features
2000–2001	Median age 4.5 years	33.3 (20 of 60)	Kamlapur, Dhaka	High fever (> 39°C) in 50%, pneumonia in 40.0% Altered mental status in 38.5%^a^ Dry pre‐Monsoon season peak
2004–2007	< 5 years	11 (249 of 2362) 16.9 in 2005, 1.3 in 2006, and 17.4 in 2007	Kamlapur, Dhaka	Out of 37 isolates A2b was in 49%, B1 in 41%, and B2 in 10%.
2004–2008	< 5 years	16 (198 of 1248)	Kamlapur, Dhaka	N/A
2009–2011	< 2 years	5 (48 of 918)^b^ 11 (41 of 378)^c^	Pallabi, Dhaka	Cough (100%), Tachypnea (100%), Reported fever (98%), Runny nose (97%), Difficulty breathing (84%), Crepitations (44%) and Ronchi (33%)
2010–2014	Median age 5 months	4 (34 of 829)	Kishoreganj, Cumilla, Bogura, Barishal	Two deaths of 34 cases. Coinfection in 15% positive samples. Cough (99%), Difficulty breathing (87%), Reasured fever (84%), Runny nose (65%), Chest indrawing (65%), Crepitation (48%), Ronchi (46%), and Wheezing (35%)
2014–2015	< 5 years	13 (26 of 200)	Dhaka, SMC	RT‐qPCR, Coinfection by *Klebsiella* spp.
2014–2016	< 6 months	5.4 (206 of 3810)	Dhaka	Peaked in January and September. Confirmation of A2b, A2c, and B1, coinfection of HPIV‐3 (*n* = 11), adenovirus (*n* = 7)
2015–2017	< 5 years	4.5 (16 of 360)	ICDDRB, Dhaka	hMPV was associated with pneumonia, RT‐qPCR, chest X‐ray
2018–2020	< 5 years	3.9 (20 of 512)	Chattogram	Coinfection of bacteria and viruses was prevalent, less frequent in people aged over 5 years, Rohingya population

a Irritability, lethargy.

^b^ Acute respiratory infections, ARI.

^c^ Pneumonia.

There are rising concerns regarding respiratory tract illness in Bangladesh, with a higher frequency of malnourished and immunocompromised populations. First, the regular hMPV outbreaks in a densely populated country with a fragile health system have been neglected till now. Second, the high prevalence of coinfection with other viruses and antibiotic‐resistant bacteria, including *K. pneumoniae* and *Mycobacterium tuberculosis*, and reinfection with other genotypes of hMPV poses a great health threat [10]. Third, there is a significant gap in molecular analysis, reporting of circulating genotypes, and analyzing the association of specific genotypes of hMPV with a particular transmission rate and disease prognosis. Fourth, after the COVID‐19 pandemic, a large number of people are suffering from reduced functionality of the respiratory system and experiencing other health complications. Fifth, the healthcare providers, including nurses and doctors, are not well trained and many of the private clinics and hospitals are harbors of antibiotic‐resistant nosocomial pathogens, including *Haemophilus influenzae* and *K. pneumoniae*. Sixth, a lack of a countrywide emergency response team to respond immediately to potential health threats and control international immigration.

## 3 The Risk Group

The symptoms associated with hMPV infections are similar to those of a mild common cold, including fever, cough, nasal congestion, and shortness of breath [1, 7]. The incubation time varies from 3 to 6 days in the majority of cases. However, the risk of severe infection and hospitalization is higher among malnourished infants, older adults, immunocompromised persons, and patients with lung disorders, asthma, and chronic obstructive pulmonary disease [7]. On top of that, the effects of hMPV infection in the lower and upper respiratory tract of COVID‐19‐infected persons remain unexplored. Outbreaks of hMPV in long‐term care facilities for young children and adults leading to fatalities have been reported numerous times [7].

## 4 Transmission in the Local Communities

**Figure 2:**
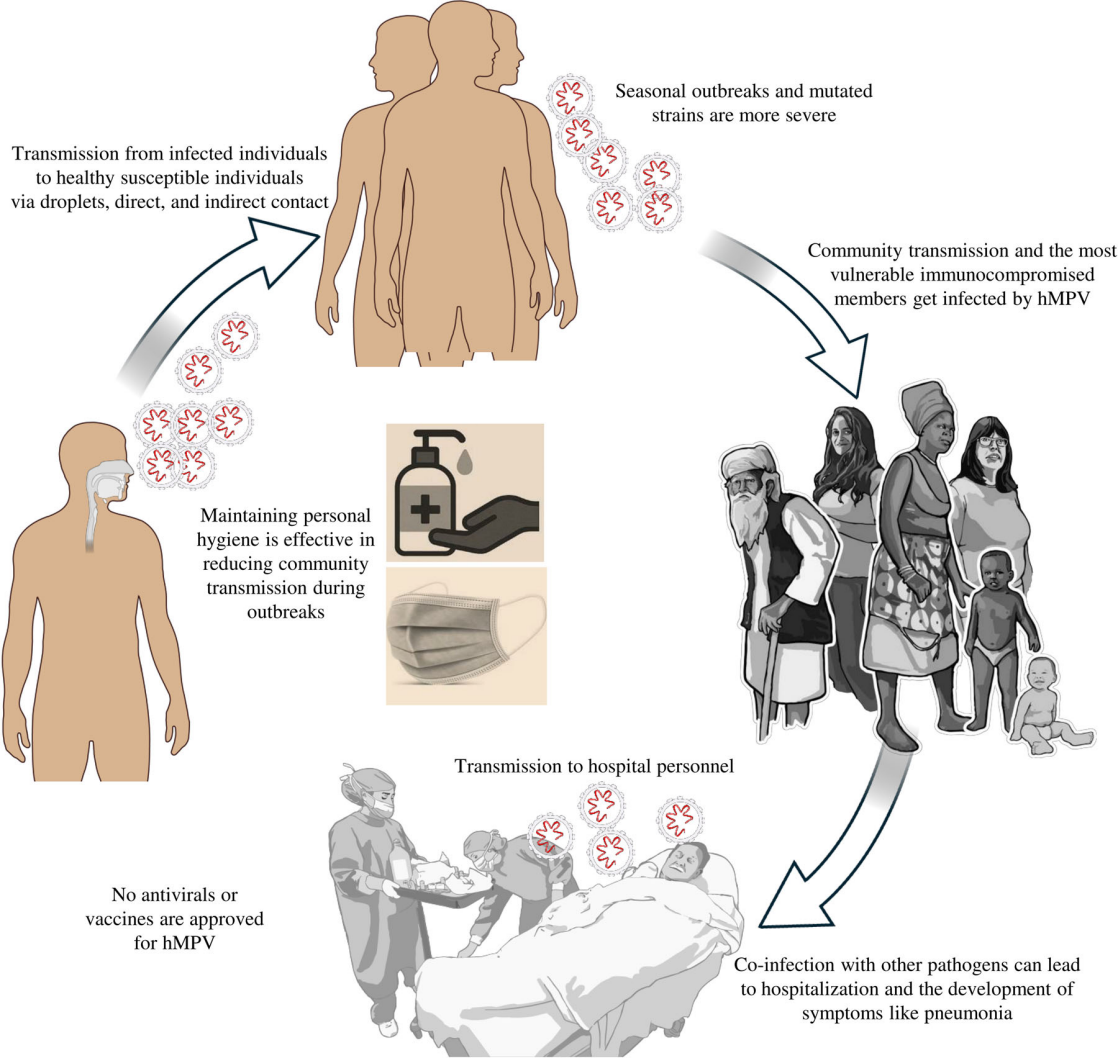
Community transmission and prevention of hMPV outbreaks.

The main ways of transmission of hMPV are well‐characterized for the circulating strains. The virus is transmitted through direct contact, contaminated fomites, respiratory droplets, and contaminated surfaces. In the local communities in Bangladesh, the practice of hygiene is poor. So, the community transmission of hMPV from an infected person to others can easily occur via coughs or sneezes and through touching contaminated surfaces, including bank notes, doorknobs, hands, nose, mouth, toys, and objects (Figure 2). Nosocomial transmission and transmission to the hospital personnel of hMPV are also common due to a higher number of less‐qualified nurses and ward boys in the hospitals.

## 5 Preventive Strategies

To minimize the potential health burden of hMPV, regular monitoring and strong countrywide surveillance involving people of all ages are required. Implementation of a rapid, accurate, and inclusive diagnosis is an urgent need. The research institutions should be funded and encouraged with more projects focusing on molecular analysis and evolutionary studies of hMPV. In addition, the high‐risk groups should be specifically identified and periodically monitored. A dedicated medical team can be prepared to focus on the management of the situation with increased transmission. Moreover, the authorities should come forward with more robust policies to address the outbreaks and increase funding for the project focusing on genotypic surveillance, organize training camps for healthcare providers, and take necessary steps to make the people aware of the exact situations of the outbreak and preventive measures such as wearing masks, maintaining personal hygiene, washing hands, avoiding crowded places and mass gathering. Though it is too early to predict the pandemic potential of hMPV outbreaks, proper health measures should be taken, focusing on the immunocompromised, malnourished, and underprivileged groups to reduce the potential health burden of future outbreaks.

## 6 Conclusions

This study reported a continuous presence of hMPV outbreaks in Bangladesh since 2000. The hMPV was one of the common pathogens among children under 5 years, hospitalized with symptoms of respiratory tract illness. Fatality among three cases of hMPV has been reported in all of these studies. Bangladesh lacks an active surveillance and outbreak monitoring system dedicated to emergency response for any major outbreaks in the future. However, the immunocompromised group of people should be provided with proper information and treatment facilities to reduce the health burden of hMPV. This study calls for more epidemiologic, genotypic, and clinical surveillance projects in the future to address the health threat of hMPV accurately.

## Author Contributions

**Nadim Sharif:** writing – original draft preparation (lead), methodology (lead), investigation (lead), conceptualization (lead), project administration (lead), writing – review and editing (equal), formal analysis (lead), data collection, data analysis, data interpretation (lead). **Shuvra Kanti Dey:** writing – review and editing (equal), formal analysis (lead), data collection, data analysis, investigation (lead), supervision (lead).

## Conflicts of Interest

The authors declare no conflicts of interest.

## Transparency Statement

The lead author Nadim Sharif affirms that this manuscript is an honest, accurate, and transparent account of the study being reported; that no important aspects of the study have been omitted; and that any discrepancies from the study as planned (and, if relevant, registered) have been explained.

## Data Availability

The data that support the findings of this study are available from the corresponding author upon reasonable request.
